# Supplementary material for: Analysis of Growth Phases of Enterotoxigenic Escherichia coli Reveals a Distinct Transition Phase before Entry into Early Stationary Phase with Shifts in Tryptophan, Fucose, and Putrescine Metabolism and Degradation of Neurotransmitter Precursors
Source: Microbiol Spectr. 2022 Jul 25;10(4):e01755-21. doi: 10.1128/spectrum.01755-21 (PMC9431495; doi:10.1128/spectrum.01755-21)
Supplement: Supplemental file 1 — Fig. S1 to S3. Download spectrum.01019-22-s0001.pdf, PDF file, 0.7 MB [file spectrum.01755-21-s0001.pdf]

Supplemental Material

Supplemental Figures

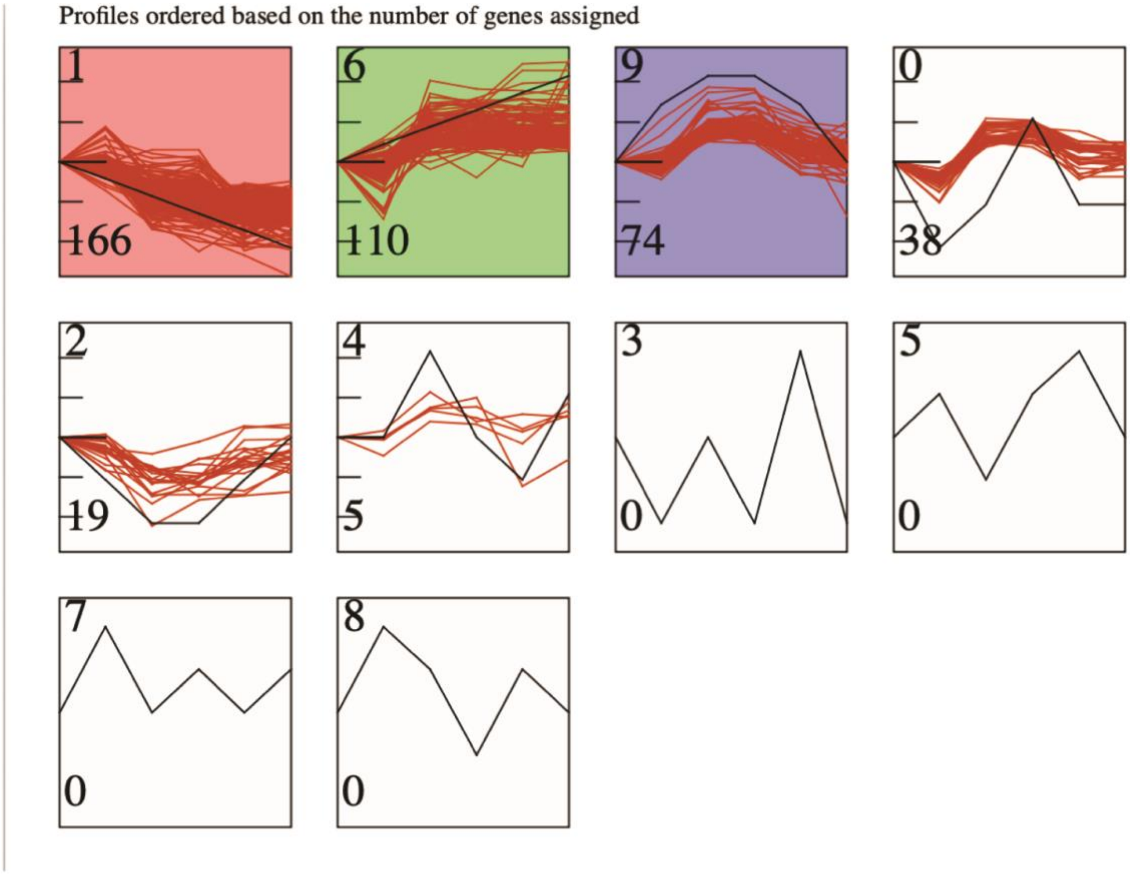

S Figure 1. Identified gene expression patterns by STEM. The profile number on the top left corner of each profile box was assigned by STEM and the number on the bottom left represents the number of genes included in the cluster. Clusters with a *p*-value > 0.05 were colored.

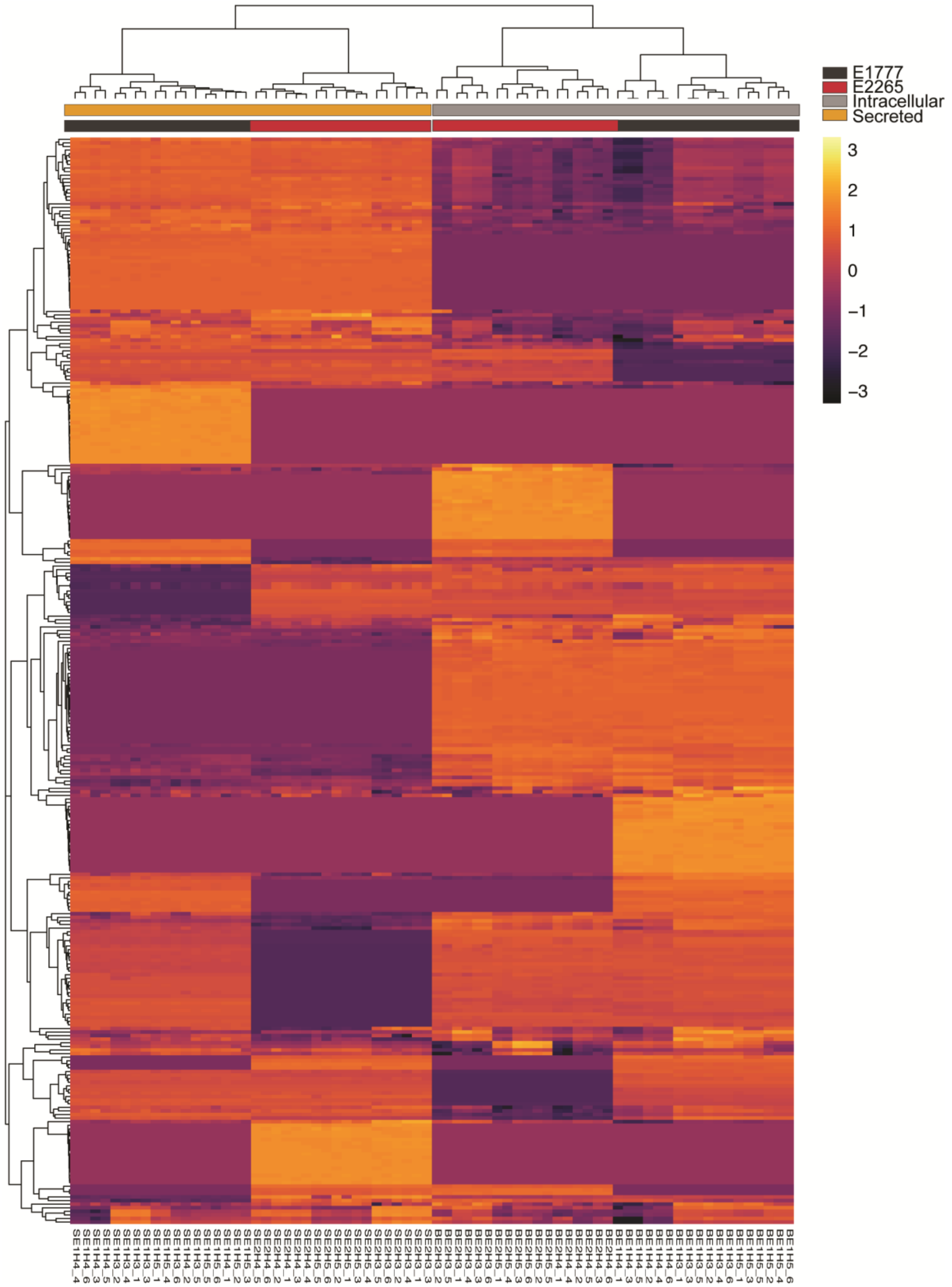

S Figure 2. Heatmap representation of the abundance of 288 intracellular and secreted metabolites detected in E2265 and E1777.

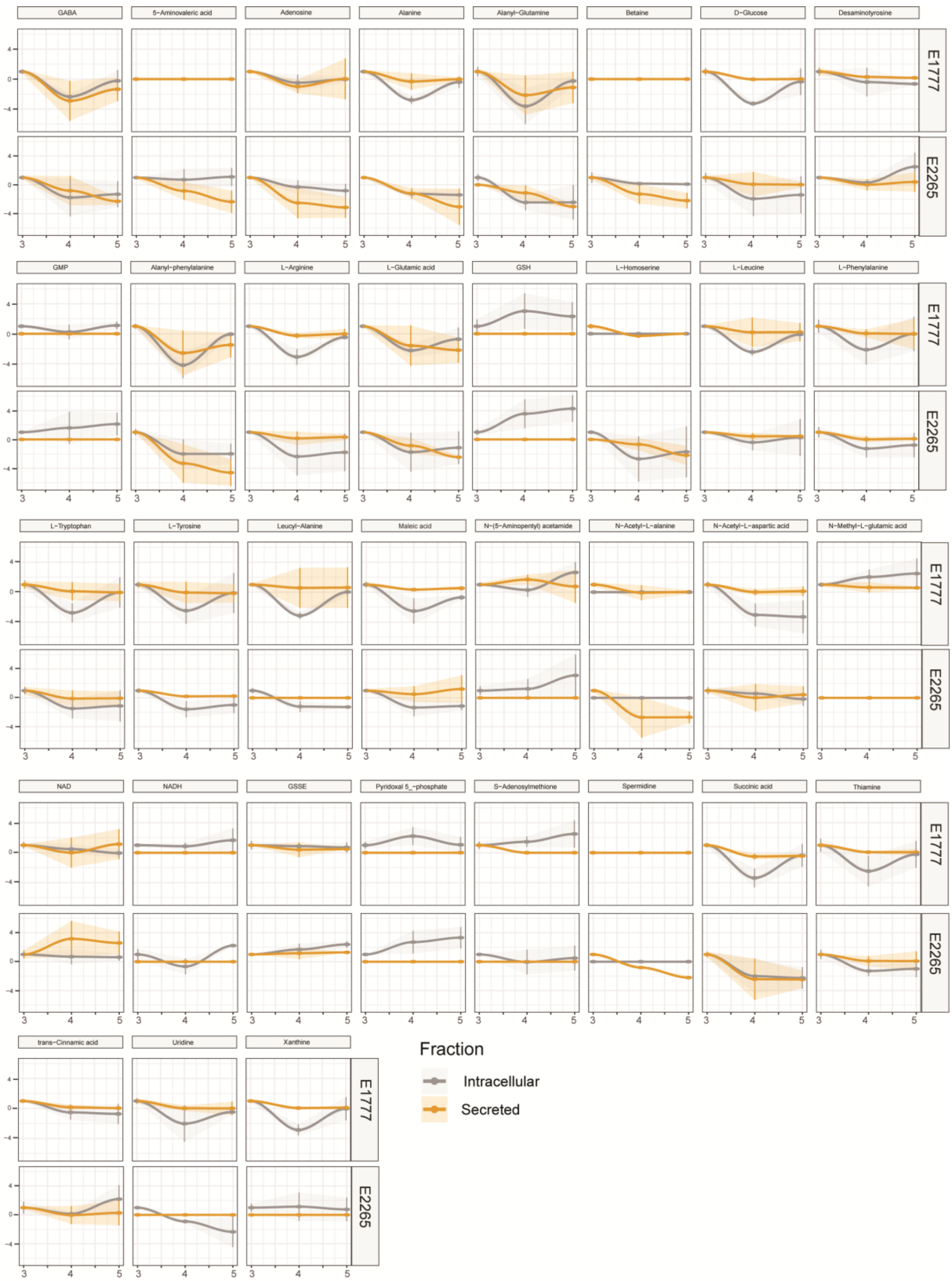

S Figure 3. Data of the concentration of all significant metabolites are represented in the serial charts. The metabolite concentration is adjusted to 3 h, and the data is presented in fold-change±SD.
